# Early continuous renal replacement therapy for postoperative patient with acute kidney injury following total pancreato-splenectomy: a case report

**DOI:** 10.1186/s13256-023-03923-z

**Published:** 2023-05-17

**Authors:** Apriliana Ratnaningrum, M. Azhari Taufik, Vera Irawany, Rudyanto Sedono

**Affiliations:** 1 Trainee of Department of Anesthesiology and Intensive Care, Dr. Cipto Mangunkusumo National Hospital, Faculty of Medicine Universitas Indonesia, Jakarta, Indonesia; 2 Department of Anesthesiology and Intensive Care, Fatmawati Center General Hospital, Jakarta, Indonesia; 3 Department of Anesthesiology and Intensive Care, Dr. Cipto Mangunkusumo National Hospital, Faculty of Medicine Universitas Indonesia, Jakarta, Indonesia

**Keywords:** Acute kidney injury, Postoperative, Case report, Early continuous renal replacement therapy, Pancreato-splenectomy

## Abstract

**Background:**

Acute kidney injury is a devastating postoperative complication. Renal replacement therapy is a treatment modality for acute kidney injury. Continuous renal replacement therapy is the treatment of choice for patients with hemodynamic instability. The main question in the management of acute kidney injury is when to initiate the renal replacement therapy. Several studies have demonstrated improvement in patients with septic acute kidney injury, following early continuous renal replacement therapy. To date, no guidelines have been established on the perfect timing to initiate continuous renal replacement therapy. In this case report, we did an early continuous renal replacement therapy as an extracorporeal therapy for blood purification and renal support.

**Case presentation:**

Our patient was a 46-year-old male of Malay ethnicity, undergoing total pancreatectomy due to a duodenal tumor. The preoperative assessment showed that the patient was high risk. Intraoperatively, massive surgical bleeding was sustained due to extensive tumor resection; thus, massive blood product transfusion was necessary. After the surgery, the patient suffered from postoperative acute kidney injury. We performed early continuous renal replacement therapy, within 24 hours after the diagnosis of acute kidney injury. Upon completion of continuous renal replacement therapy, the patient’s condition improved, and he was discharged from the intensive care unit on the sixth postoperative day.

**Conclusion:**

The timing for the initiation of renal replacement therapy remains controversial. It is clear that the “conventional criteria” for initiating renal replacement therapy need correction. We found that early continuous renal replacement therapy initiated in less than 24 hour after the postoperative acute kidney injury diagnosis gave our patient survival benefit.

## Background

Acute kidney injury (AKI) is a frequent problem in critically ill patients and those following major abdominal surgery. AKI is associated with prolonged hospital stay, increased risk of nosocomial infections, and significant cost burden [1, 2]. The etiology of postoperative AKI after major abdominal surgery is complex, including the effects of fluid loss or hypovolemia, neurohormonal responses to anesthesia and surgical trauma, damage-associated molecular pattern (DAMP)-induced inflammation, and intraabdominal pressure [3].

The management of AKI after a major abdominal surgery includes hemodynamic stabilization, fluid balance control, nephrotoxin removal, and renal replacement therapy (RRT). Evidences support an early RRT in this setting [3, 4]. Several metaanalyses have shown that early initiation of RRT yields survival benefits and recommend early RRT for patients with AKI following cardiac surgery [5]. Shiao et al. conducted a prospective observational study on 98 patients with AKI who required RRT after major abdominal surgery [6].

## Case presentation

We report a case of 46-year-old male complaining of abdominal pain and obstructive jaundice. He was referred to the digestive surgeon at Fatmawati Central General Hospital. The physical examination revealed that the abdomen was distended with palpably enlarged spleen and liver. An abdominal contrast computed tomography (CT) scan found lobulated masses in the duodenal and ampulla of vater projection. Surgical intervention was decided and the patient was admitted to the intensive care unit (ICU) after a total pancreato-splenectomy due to a stage IIIb duodenal tumor.

During the preoperative assessment, we concluded that this patient was high risk due to anemia, obstructive jaundice, and decreased liver function. Intraoperatively, massive surgical bleeding (2500 cc) was sustained due to extensive tumor resection, and thus massive blood product transfusion was necessary.

Upon arrival at the ICU, our patient had decreased urine output. Therefore, we had to increase the vasopressor dose to 0.3 µg/kg/minute to maintain the mean arterial pressure above 65 mmHg and systolic blood pressure above 95 mmHg. Laboratory examination revealed a lactate level of 5.8 mmol/L, C-reactive protein (CRP) level of 30 mg/dL, white blood cell counts of 26,700/μL, and procalcitonin level of > 32 ng/mL. These findings showed that the patient had a systemic inflammation response syndrome (SIRS) due to the surgery. Furthermore, 12 hours after the surgery, he showed signs of AKI, including urine production of < 0.3 cc/kg/hour. Hyperglycemia was another problem observed in the patient, with the highest glucose level recorded at 511 mg/dL within 3 hours after the surgery. Glucose management therapy was started with an insulin infusion drip of up to 7 IU/hour.

**Fig. 1:**
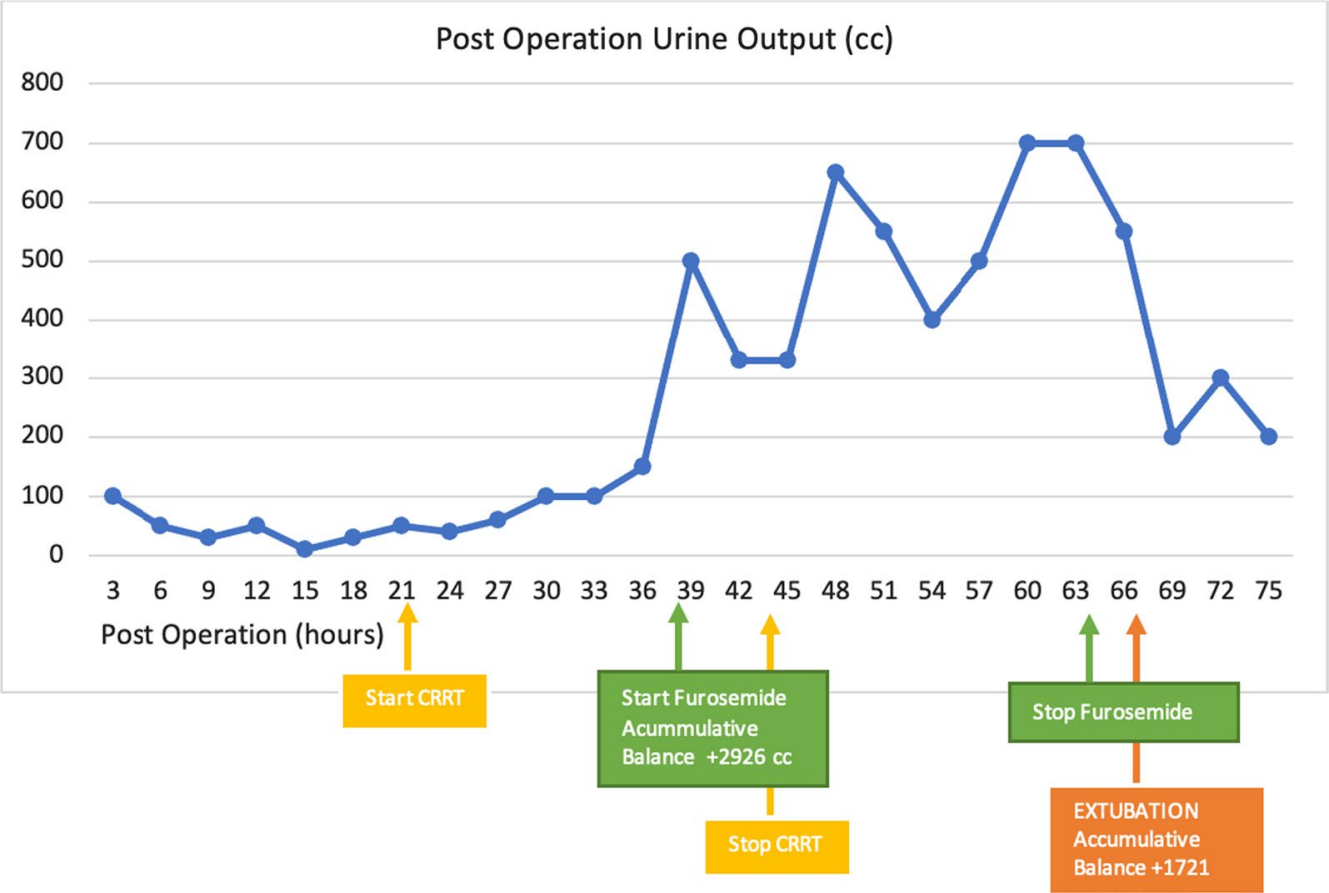
Postoperative urine output (cc)

We decided to initiate early continuous renal replacement therapy (CRRT) for this patient. The CRRT was initiated within 24 hour following the diagnosis of postoperative AKI on the basis of Kidney Disease Improving Global Outcome (KDIGO) criteria stage 2. CRRT was conducted using the continuous veno-venous hemofiltration (CVVH) method. The effluent dose was 27 cc/kg/hour with 0 cc fluid removal. After 3 hours of CVVH initiation, the patient showed signs of improvement. His urine output increased to 0.5–0.6 cc/kg/hour, and the vasopressor dose was quickly tapered. We decided to administer furosemide infusion within 18 hours after CVVH, and the patient’s urine output increased to 1–4 cc/kg/hour (Fig. 1).

**Fig. 2:**
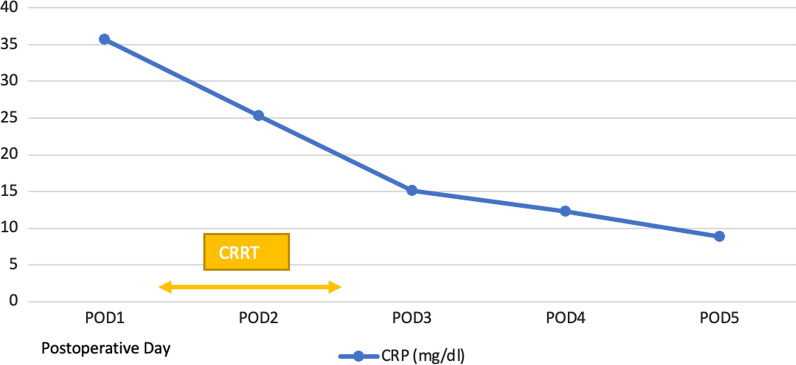
Postoperative CRP level (mg/dL)

**Fig. 3:**
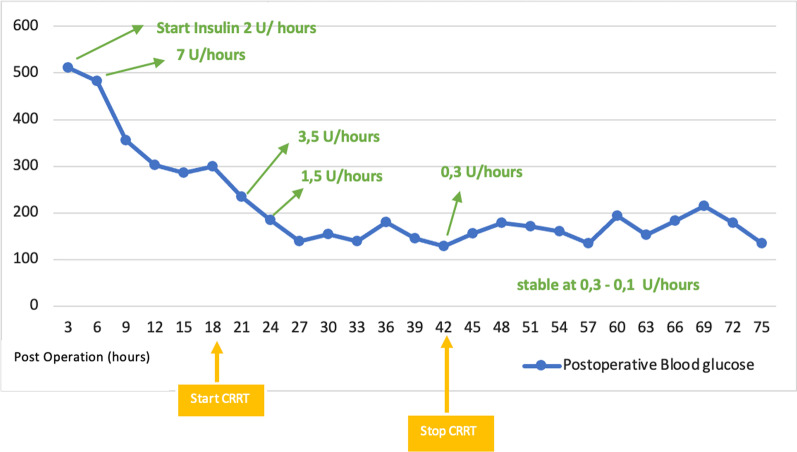
Postoperative blood glucose level (mg/dL)

The patient’s inflammation was assessed on the basis of his CRP levels. On day 1 after the surgery, the patient’s CRP level was 35 mg/dL. Following the initiation of CVVH on the third postoperative day, his CRP level decreased significantly to 15.1 mg/dL. The inflammation was significantly reduced as the CVVH continued (Fig. 2). After the initiation of CVVH, the patient’s blood glucose was controlled and the insulin infusion drip rate was reduced to 0.3 U/hour (Fig. 3).

**Fig. 4:**
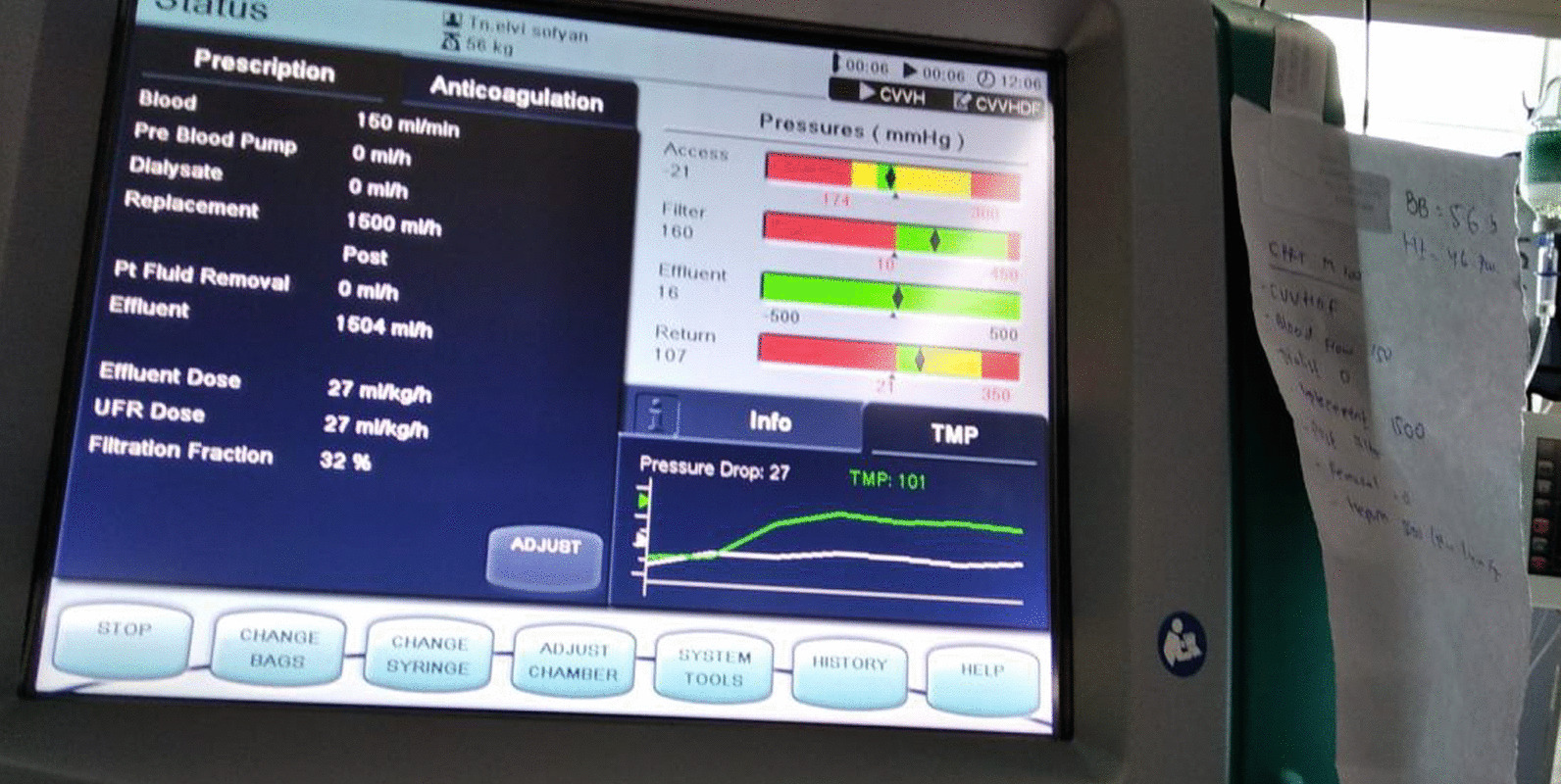
CRRT prescription

On the second postoperative day, the patient’s white blood cell count and procalcitonin level increased, indicating an ongoing infection. We changed the antibiotic course and administered an empirical broad-spectrum antibiotic instead, with 3 × 2 g intravenous meropenem. We observed a good clinical response after the CRRT was initiated (Fig. 4). Urine output was increasing and we could reduce the norepinephrine dose. The CRRT was stopped on the third postoperative day and furosemide was started at 1 mg/hour for renal support.

**Fig. 5:**
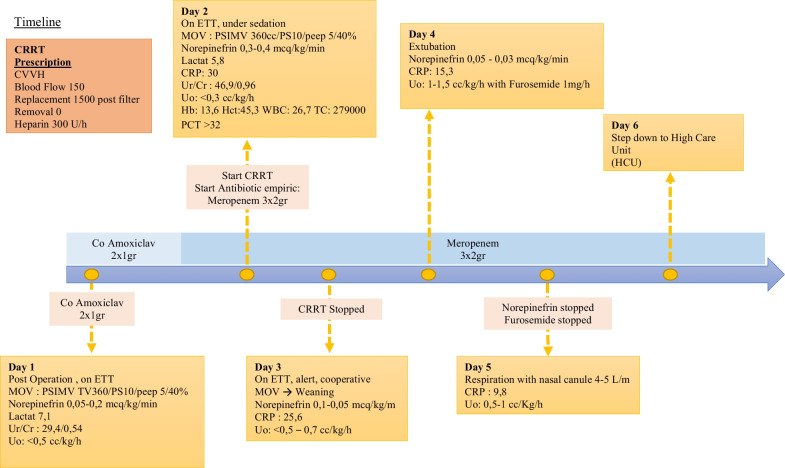
Patient’s timeline since ICU admission

On the fourth postoperative day, the patient was alert and cooperative with minimal ventilatory and hemodynamic support. The patient was extubated and respiratory support was provided with a nasal cannula (Fig. 5).

## Discussion

Preoperative risk assessment revealed that our patient was high risk due to anemia, obstructive jaundice, and decreased liver function. During the surgery, he experienced massive bleeding because of the extensive surgical tumor resection. Intraoperative massive bleeding led to hypovolemia and massive blood product transfusion. This condition was a procedure-related factor for AKI following major abdominal surgery. In the ICU, the patient’s urine output was found to be decreased and the vasopressor dose had to be increased. These showed that the patient was in a severe catecholamine storm, due to releasing inflammatory mediators, and might suffer from organ failures. A strategy was necessary for blood purification and clearance of the inflammatory mediators, which might have minimized the renal injury and provided organ support [6–10] (Table 1).

**Table 1:** Staging of AKI from KDIGO 2012 Clinical Practice Guidelines [12]

Stage	Serum creatinine	Urine output
1	1.5–1.9 times baseline OR ≥ 0.3 mg/dL (≥ 2.65 μmol/L) increase	< 0.5 mL/kg/hour for 6–12 h
2	2.0–2.9 times baseline	< 0.5 mL/kg/hour for ≥ 12 h
3	3.0 times baseline OR Increase in serum creatinine to ≥ 4.0 mg/dL (≥ 353.6 μmol/L) OR Initiation of renal replacement therapy OR, in patients < 18 years, decrease in eGFR to < 35 mL/minute per 1.73 m^2^	< 0.3 mL/kg/hour for ≥ 24 hour OR anuria for ≥ 12 hour

The patient also experienced hyperglycemia. Hyperglycemia related to inflammation and hypermetabolic stress response commonly occurs in postoperative patients. In this patient, the loss of pancreatic parenchymal tissue from the pancreatic resection also worsened the disruption of glucose homeostasis, known as pancreatogenic diabetes [9]. The presence of diabetes could worsen this serious inflammatory condition, leading to organ failure [11].

CRRT was initiated as an extracorporeal therapy to suppress circulatory inflammatory mediators and boost renal support with minimal hemodynamic disruption. CVVH (continous veno-venous hemofiltration) was initiated within 24 hours after the diagnosis of postoperative AKI was established on the basis of KDIGO criteria stage 2 [12].

In this case, the purpose of early initiation of RRT was to act as an immunomodulator, remove inflammatory mediators, and prevent organ failure. Additionally, early RRT aimed to prevent severe electrolyte and acid–base imbalances, balance the glucose homeostasis, prevent uremic complications, manage volume overload, and prevent unnecessary or excessive diuretic exposure [1–4].

The clinical decision of when to initiate RRT remains controversial. However, early initiation of RRT in patients with severe sepsis might be beneficial. Although early RRT is not associated with any particular benefits, avoiding or delaying RRT is associated with higher mortality and increased hospital/ICU length of stay [13].

## Conclusion

Extracorporeal blood purification, which aimed to remove cytokines, was necessary for our high-risk postoperative patient, who suffered from a hyperdynamic condition due to circulating inflammatory mediators. Early CRRT provides supportive therapy until the kidneys can continue functioning independently. The timing for RRT initiation remains controversial. It is clear that the “conventional criteria” for initiating RRT need correction. It has been suggested that early initiation of RRT in patients with septic AKI may improve outcomes. We found that early CRRT, initiated within 24 hours after the diagnosis of postoperative AKI, gave our patient survival benefit.

## Data Availability

Not applicable.

## Abbreviations

AKI: Acute kidney injury
CRP: C-reactive protein
CRRT: Continuous renal replacement therapy
CVVH: Continuous veno-venous hemofiltration
DAMP: Damage-associated molecular pattern
ICU: Intensive care unit
RRT: Renal replacement therapy

## References

[1] Romagnoli S, Zagli G, Tuccinardi G, Tofani L, Chelazzi C, Villa G (2016). Postoperative acute kidney injury in high-risk patients undergoing major abdominal surgery. J Crit Care.

[2] Hoste EA, Bagshaw SM, Bellomo R, Cely CM, Colman R, Cruz DN (2015). Epidemiology of acute kidney injury in critically ill patients: the multinational AKI-EPI study. Intensive Care Med.

[3] Gameiro J, Fonseca JA, Marques F, Lopes JA (2020). Management of acute kidney injury following major abdominal surgery: a contemporary review. J Clin Med.

[4] Goren O, Levy A, Cattan A, Lahat G, Matot I (2017). Acute kidney injury in pancreatic surgery; association with urine output and intraoperative fluid administration. Am J Surg.

[5] Zou H, Hong Q, Xu G (2017). Early versus late initiation of renal replacement therapy impacts mortality in patients with acute kidney injury post cardiac surgery: a meta-analysis. Crit Care.

[6] Shiao CC, Wu VC, Li WY, Lin YF, Hu FC, Young GH, National Taiwan University Surgical Intensive Care Unit-Associated Renal Failure Study Group (2009). Late initiation of renal replacement therapy is associated with worse outcomes in acute kidney injury after major abdominal surgery. Crit Care.

[7] Ronco C, Ricci Z, De Backer D, Kellum JA, Taccone FS, Joannidis M (2015). Renal replacement therapy in acute kidney injury: controversy and consensus. Crit Care.

[8] Shiao CC, Wu V, Li WY, Lin YF, Hu FC, Young GH (2009). Late initiation of renal replacement therapy is associated with worse outcomes in acute kidney injury after major abdominal surgery. Crit Care.

[9] Maeda H, Hanazaki K (2011). Pancreatogenic diabetes after pancreatic resection. Pancreatology.

[10] Gumbert SD, Kork F, Jackson ML, Vanga N, Ghebremichael SJ, Wang CY (2020). Perioperative acute kidney injury. Anesthesiology.

[11] Lee KW, Devaraj NK, Ching SM, Veettil SK, Hoo FK, Deuraseh I, Soo MJ (2021). Effect of SGLT-2 inhibitors on non-alcoholic fatty liver disease among patients with type 2 diabetes mellitus: systematic review with meta-analysis and trial sequential analysis of randomized clinical trials. Oman Med J.

[12] Walther CP, Podoll AS, Finkel KW (2014). Summary of clinical practice guidelines for acute kidney injury. Hosp Pract (1995).

[13] Castro I, Relvas M, Gameiro J, Lopes JA, Monteiro-Soares M, Coentrão L (2022). The impact of early versus late initiation of renal replacement therapy in critically ill patients with acute kidney injury on mortality and clinical outcomes: a meta-analysis. Clin Kidney J.

